# Effects of acute physical activity on executive functions requiring inhibition among children with attention-deficit hyperactivity disorder

**DOI:** 10.1192/j.eurpsy.2022.387

**Published:** 2022-09-01

**Authors:** M. Miklós, D. Komáromy, J. Futó, J. Balazs

**Affiliations:** 1 Eötvös Loránd University, Institute Of Psychology, Department Of Developmental And Clinical Child Psychology, Budapest, Hungary; 2 Pedagogical Assistance Services, Budapest, Hungary; 3 Vrije Universiteit Amsterdam, Faculty Of Behavioural And Movement Sciences, Amsterdam, Netherlands; 4 Universiteit van Amsterdam, Faculty Of Social And Behavioral Sciences, Amsterdam, Netherlands; 5 Bjørknes University College, Oslo, Norway

**Keywords:** ADHD, executive function, inhibition, children

## Abstract

**Introduction:**

In recent years, physical activity as a potential intervention for attention-deficit hyperactivity disorder (ADHD) became into the focus of researchers, however the results are conflicting.

**Objectives:**

Our aim was to investigate the effect of acute moderate physical activity on executive functions requiring inhibition.

**Methods:**

The study included 50 treatment-naïve ADHD children, 50 medicated children with ADHD and 50 typically developing children, aged 6–12 years. To diagnose ADHD, we applied the Mini International Neuropsychiatric Interview for Children and Adolescents. To measure executive functions, the pediatric version of the Test of Attentional Performance (KiTAP) was used. Half of the children in each study group participated in a 20-minute, moderately intense exercise while watching a cartoon video. In the control intervention, the other half of the children from all three study groups watched the same cartoon video in a sitting position for 20 min.

**Results:**

Regarding distractibility, flexibility and inhibition, physical activity had a significant positive effect on two of 10 parameters (number of total errors and errors when distractor was presented, both in the distractibility task) in the treatment-naïve ADHD group.

**Conclusions:**

Our results suggest that moderate acute physical activity has some significant positive effects on certain executive function parameters among children with ADHD. Future studies should consider determining the optimal form, intensity, and duration of physical activity to become a potential adjunctive intervention for children diagnosed with ADHD.

**Disclosure:**

No significant relationships.

